# Challenges with PrEP Uptake and Adherence Among Gay, Bisexual, and Other Men Who Have Sex with Men in Kisumu, Kenya

**DOI:** 10.1007/s10461-022-03860-w

**Published:** 2022-10-11

**Authors:** Susan M. Graham, Duncan O. Okall, Supriya D. Mehta, Eve Obondi, George Ng’ety, Elijah Ochieng, Laura Jadwin-Cakmak, K. Rivet Amico, Gary W. Harper, Robert C. Bailey, Fredrick O. Otieno

**Affiliations:** 1 Departments of Global Health, Medicine, and Epidemiology, University of Washington, 325 Ninth Avenue, Box 139909, Seattle, WA 98104 USA; 2 Nyanza Reproductive Health Society, Kisumu, Kenya; 3 Division of Epidemiology and Biostatistics, University of Illinois at Chicago, Chicago, IL USA; 4 Salina Youth Initiative, Kisumu, Kenya; 5 Department of Health Behavior & Health Education, University of Michigan, Ann Arbor, MI USA

**Keywords:** HIV, Pre-exposure prophylaxis, Adherence, Men who have sex with men, Kenya, Tenofovir disoproxil fumarate

## Abstract

Data on challenges with pre-exposure prophylaxis (PrEP) uptake and adherence among Kenyan gay, bisexual, and other men who have sex with men (GBMSM) are limited. In this mixed-methods sequential explanatory design study, our quantitative phase followed 157 at-risk, HIV-negative GBMSM who accepted PrEP and enrolled in a cohort with 12-month follow-up. Stored dried blood spots collected at two intervals were batch tested for tenofovir diphosphate (TFV-DP) concentrations at study end. Despite high self-reported adherence, only 14.6% of individuals had protective TFV-DP levels at any visit. Protective TFV-DP levels were positively associated with injection drug use and a self-assessed moderate risk of acquiring HIV, and negatively associated with time since enrolment. In our subsequent qualitative phase, an intensive workshop was conducted with the GBMSM community to identify barriers and facilitators to PrEP uptake and adherence. These data revealed numerous challenges with traditional PrEP programs that must be addressed through community collaborations.

**Supplementary Information:**

The online version contains supplementary material available at 10.1007/s10461-022-03860-w.

## Resumen

La evidencia respecto a desafíos existentes con aceptación y adherencia de la profilaxis previa a la exposición (PrEP) de VIH, entre los hombres homosexuales, bisexuales y otros hombres que tienen sexo con hombres (GBMSM) en Kenia es limitada. Condujimos un estudio de métodos mixtos y diseño explicativo secuencial. En la fase cuantitativa seguimos a 157 GBMSM VIH-negativos en riesgo que aceptaron PrEP y se inscribieron en una cohorte con un seguimiento de 12 meses. Analizamos, por lotes y al final del estudio, gotas de sangre seca recolectada a dos intervalos de tiempo y previamente almacenada, para determinar las concentraciones de difosfato de tenofovir (TFV-DP). A pesar de la alta adherencia autoinformada, solo el 14,6% de las personas tenían niveles protectores de TFV-DP en alguna visita. Los niveles protectores de TFV-DP se asociaron positivamente con el uso de drogas inyectables y un riesgo moderado autoevaluado de contraer el VIH, y negativamente con el tiempo transcurrido desde la inscripción. En la fase cualitativa posterior, conversamos con GBMSM de la comunidad para identificar las barreras y los facilitadores para la concientización, aceptación, adherencia y retención a PrEP. Estos datos revelaron numerosos desafíos con los programas tradicionales de PrEP que deben abordarse mediante colaboraciones comunitarias.

## Introduction

The HIV pandemic has disproportionally impacted gay, bisexual, and other men who have sex with men (GBMSM) globally, and efforts to prevent new infections in this population are of paramount importance [1, 2]. The potential impact of daily pre-exposure prophylaxis (PrEP) with tenofovir-emtricitabine on at-risk populations was demonstrated in the 6-country iPrEx trial, which enrolled 2,499 GBMSM and transgender women and reported a 44% reduction in HIV acquisition [3], with up to 90% effectiveness among participants who had intracellular tenofovir diphosphate (TFV-DP) levels > 16 fmol per million in stored peripheral blood mononuclear cells [4]. Overall, PrEP studies have demonstrated consistent effectiveness at reducing HIV infection risk for rectal exposure (relative risk [RR] 0.34, 95% confidence interval [CI] 0.15–0.80) [5]. However, post-trial analyses have repeatedly shown that PrEP efficacy is closely associated with adherence as measured by drug detection in serum or tissues [4, 6]. In the STRAND trial, where doses were directly observed, blood levels of TFV-DP achieved 99% protection with 7 doses per week, while ≥ 4 doses per week were estimated to provide 96% protection [4].

In 2014, the WHO released strong recommendations to include PrEP as an option in combination prevention packages for GBMSM [7]. In the same year, Kenya incorporated PrEP for key populations into its HIV Prevention Revolution Road Map [8]. Soon afterwards, the Kenya Pharmacy and Poisons Board approved PrEP for HIV prevention [9]. In 2017, Kenya officially adopted PrEP as part of combination HIV prevention for individuals at substantial ongoing risk, specifically including GBMSM [10].

Prior to the WHO recommendation for PrEP, evidence about PrEP uptake and adherence among GBMSM in East Africa was limited. One small Kenyan trial conducted in 2009–2010 compared daily PrEP to an intermittent regimen (a fixed dose on Mondays and Fridays and a post-coital dose within 2 h after sex, not to exceed 1 dose per day), enrolling 62 GBMSM and demonstrating high acceptability [11, 12]. Median adherence to daily PrEP in this trial, using electronic monitoring, was 80% of prescribed doses [13]. Additionally, qualitative interviews conducted in Kisumu and Nairobi counties in 2013–2014 showed strong interest among GBMSM participants, with 83% saying they were willing to take daily oral PrEP [14].

Since that time, limited data have emerged about PrEP use by GBMSM participating in research on the Kenyan coast; these studies have shown relatively high levels of uptake (69.7–82.4% of those eligible) [15, 16], but low adherence and low continuation of PrEP [16–18]. Data on PrEP use and adherence by African GBMSM, including biomarker-based adherence assessments and information regarding barriers and facilitators as perceived by African GBMSM individuals and their communities, are urgently needed. Such studies would benefit from using a mixed-methods sequential explanatory design, in which qualitative data are collected after a quantitative study in order to explain, or elaborate on, the quantitative results.

Our aims in the current study were to: (1) evaluate PrEP uptake, self-reported adherence, and biomarker-based adherence in a cohort of GBMSM at high risk for HIV in western Kenya, followed for 12 months after acceptance of PrEP services; (2) identify factors associated with protective TFV-DP levels in dried blood spots (DBS); and (3) present data on the cohort’s high self-reported adherence but low TFV-DP levels to community members and explore barriers and facilitators to PrEP uptake and adherence in a charrette format.

## Methods

### PrEP Cohort

In October 2017, we established an observational cohort called *Anza Mapema Mbili* (“Start Early Two”) to evaluate PrEP uptake and adherence among GBMSM in Kisumu, Kenya over 12 months of follow-up. Men were screened for cohort eligibility if they had reported high-risk sexual behavior while participating in an earlier cohort study (2015–2017) called *Anza Mapema* (Kiswahili for “Start Early”), which followed 636 HIV-negative adult GBMSM for 12 months before PrEP became available [19]. Men who had completed *Anza Mapema* were eligible for *Anza Mapema Mbili* screening if they were HIV-negative at screening and reported any of the following in the past 3 months at their last *Anza Mapema* study visit: three or more male sex partners, condomless anal sex with a male partner who was living with HIV or of unknown HIV status, treatment for a sexually transmitted infection (STI), engagement in transactional sex, or injection drug use (IDU). These same risk factors were required for eligibility at study screening. In addition, men had to be interested in starting PrEP, live in Kisumu, and be willing to attend *Anza Mapema Mbili* study visits for the 12-month follow-up period. Screening outreach included information on the availability of a new pill that could prevent HIV infection if taken consistently. In addition, educational sessions on PrEP were held at the *Anza Mapema* clinic and at community-based organizations serving GBMSM in the study area, to increase PrEP awareness and encourage participation. Enrollment in *Anza Mapema Mbili* was completed in December 2017, and follow-up ended in February 2019.

### Cohort Procedures

All participants were offered HIV prevention services including health education, condoms, and lubricants at baseline and follow-up visits. At the baseline visit, a study counselor and clinician separately discussed PrEP with each participant, and those who accepted to start PrEP were provided with a 30-day PrEP supply. A review of PrEP adherence and side effects took place at week 2. Afterwards, study visits occurred at months 1, 2 and 3; then quarterly for 1 year. At each monthly or quarterly visit, HIV testing and counseling were provided according to Kenyan national guidelines [20], followed by PrEP adherence counseling. Study counselors had received a 2-day training on the basics of motivational interviewing prior to the study launch [21, 22], focusing on an approach to explore what made it harder or easier to take PrEP pills daily. Of note, clinicians and counselors were not members of the GBMSM community, but had worked for many years with this community and collaborated with GBMSM peer outreach workers who supported recruitment and retention [23].

At baseline and each quarterly visit, participants underwent an audio computer-assisted self-interview (ACASI) in English, Kiswahili, or DhoLuo. This questionnaire captured data on sociodemographic factors at baseline and sexual practices, substance use, mental health, and PrEP adherence during follow-up, with some measures collected quarterly and some every 6 months. Study staff were available in case of technical problems or questions. Participants who reported hazardous or harmful drinking or moderate to severe abuse of other substances were engaged for supportive counselling at the *Anza Mapema* clinics or referred to local services.

After the ACASI, a standardized medical history and physical examination were performed at each visit, with review for syndromic STIs and any PrEP-related side effects (if applicable) before refills were provided. HIV testing was performed at each visit, and participants who tested positive for HIV discontinued PrEP and were linked to immediate HIV care and treatment. HIV-negative participants who chose to discontinue PrEP remained in follow-up and continued to receive HIV prevention services, including quarterly HIV testing. Participants who missed visits were contacted by telephone up to three times, and if unavailable were traced in person. Participants received 500 Kenyan shillings (approximately $5.00) for transportation and time at each study visit, per local research guidelines.

### Cohort Measures

Baseline characteristics included: age, education, occupation, marital status, and whether the participant lived with a male partner. Sexual practices assessed included sex with male and female partners, number of male and female partners, transactional sex, condom use during anal intercourse with male partners, usual sexual position, injection drug use, and needle sharing. Validated instruments were used to assess alcohol use (Alcohol Use Disorder Identification Test [AUDIT]) [24], use of substances other than alcohol and tobacco (Drug Abuse Screening Test 10 [DAST-10]) [25], and depressive symptoms (Patient Health Questionnaire 9 [PHQ-9]) [26]. Social support was assessed using the Medical Outcomes Survey-Social Support Survey (MOS-SSS) [27]. Childhood sexual abuse was assessed [28], as well as experiences of verbal abuse, physical abuse, forced sex, or threats in the last 3 months [29]. Finally, disclosure of same-sex relationships to others (family, friends, coworkers or fellow students, the public) was assessed.

### PrEP Adherence

PrEP adherence assessments at each study visit included a question on when PrEP was last taken, three self-report items (number of days missed in past month, self-rated ability to take PrEP as prescribed, and how often PrEP was taken) [30, 31], and a visual analog scale [32]. DBS were collected and stored until shipment to the Anderson laboratory in Denver, Colorado, USA for testing. Target collection visits per protocol were at month 3 and month 9, but if the participant missed either visit or the sample collection was missed for any reason, collection was done at the next available opportunity. Detection of TFV-DP was defined as a level ≥ 25 fmol/punch. Protective TFV-DP levels were defined as ≥ 700 fmol/punch, compatible with ≥ 4 doses per week. TFV-DP levels were also categorized as follows: undetectable to 349 (< 2 doses per week), 350–699 (2–3 doses per week), 700–1249 (4–6 doses per week), and 1250 + fmol/punch (7 doses per week) [4].

### Statistical Analysis

A flow diagram was used to present data on cohort screening, enrolment, PrEP uptake, visit attendance, HIV seroconversions, and DBS collection timepoints. Descriptive statistics were used to summarize demographic, behavioral, and mental health characteristics of cohort participants at baseline. In addition, we created a descriptive table of sexual risk behavior and adherence over all follow-up visits and tested for trend across visits using Cuzick’s nonparametric test for trend (Stata’s *nptrend* command). For this table, a composite measure of HIV risk was created to indicate when any of the following was present: two or more partners (male or female), transactional sex, inconsistent condom use with male partners, injection drug use, or needle sharing. Of note, 2017 estimates of HIV prevalence in Kisumu County among adults aged 15–49 was 15.0% for males and 17.4% for females [33].

To identify participant characteristics associated with protective levels of TFV-DP across participants and study visits, generalized estimating equations (GEE) with a logit link, exchangeable correlation matrix, and robust standard errors were used. All available DBS data were included (i.e., multiple TFV-DP outcomes per participant were possible). Variables significant in bivariable analysis at *P* < 0.10 and time since enrolment a priori were included in multivariable modelling. Where two variables met criteria but were collinear (i.e., sex with a female and number of female partners), the more significant variable was retained. Separately, we analyzed associations between each self-reported adherence measure and protective levels of TFV-DP using GEE. We also calculated Spearman rank correlation coefficients between the self-reported adherence measures and TFV-DP levels as a continuous measure. *P* values were 2-sided, with values < 0.05 considered statistically significant. Data were analyzed using Stata 15.0 (StataCorp LLC, College Station, Texas, USA).

### Community Charrette

After the results of DBS testing became available and showed much lower TFV-DP levels than expected, members of the research team who were embarking on a project to develop a PrEP support intervention convened a community feedback meeting. This half-day community meeting was designed as an intensive workshop or “charrette” [34] in which community stakeholders (members of local community-based organizations serving GBMSM, some of whom had participated in the study) reviewed results of the *Anza Mapema Mbili* study and identified and discussed barriers and facilitators of PrEP uptake and adherence. At the beginning of the charrette, the research team presented results of DBS testing for the *Anza Mapema Mbili* study, as well as an overview of the planned work to design and test an intervention to improve sexual health. We posed the question to the group of why they thought GBMSM were not taking full advantage of PrEP for HIV prevention. Participants then divided into four groups that were co-facilitated by a research team member and a GBMSM community leader, and were asked to identify barriers and facilitators to (1) PrEP uptake (defined as things that hinder or help GBMSM initiate PrEP); and (2) PrEP adherence (defined as things that hinder or help GBMSM take PrEP as prescribed). Barriers and facilitators were recorded by one member of each group on flipchart paper. At the end of the day, all four groups came together for presentation and discussion of their results, and flipcharts were modified to reflect additional points raised in the discussion. All flipchart materials were retained and subsequently summarized, with barriers and facilitators grouped into categories and synthesized in table format. Community members who attended the charrette were considered critical key informants representing both the GBMSM community and the HIV service provider community (e.g., two community groups involved in PrEP scale-up at the time) and were fed and compensated 750 Kenyan schilling (about $7.50) for their time and transportation costs. The day after this community meeting, the five peer leaders from the GBMSM community who co-facilitated the charrette met with the research team to debrief and discuss implications of the community meeting results for the development of a PrEP support intervention.

### Qualitative Analysis

We utilized a thematic approach to data analysis to explore the results of the community charrette [35, 36]. Thematic analysis is a versatile and flexible research method that can be applied to a range of data different types and was appropriate to our objective of creating a broad phenomenological understanding of the GBMSM community’s views on PrEP use, including why it should or shout not be initiated (as captured by the PrEP uptake barriers and facilitators) and how it should be used once started (as captured by the PrEP adherence barriers and facilitators) [35, 36]. This approach focused on the identification of broad themes within which the charrette data could be grouped and summarized for each step of the PrEP cascade, generating a thematic “map” to show patterns in the semantic content elicited. Themes included in this table were further analyzed and interpreted in the context of the peer debrief meeting, in an effort to theorize the significance of patterns and the broader meanings and implications of the data, including potential solutions for the barriers identified as discussed by the peer leaders.

## Results

**Fig. 1:**
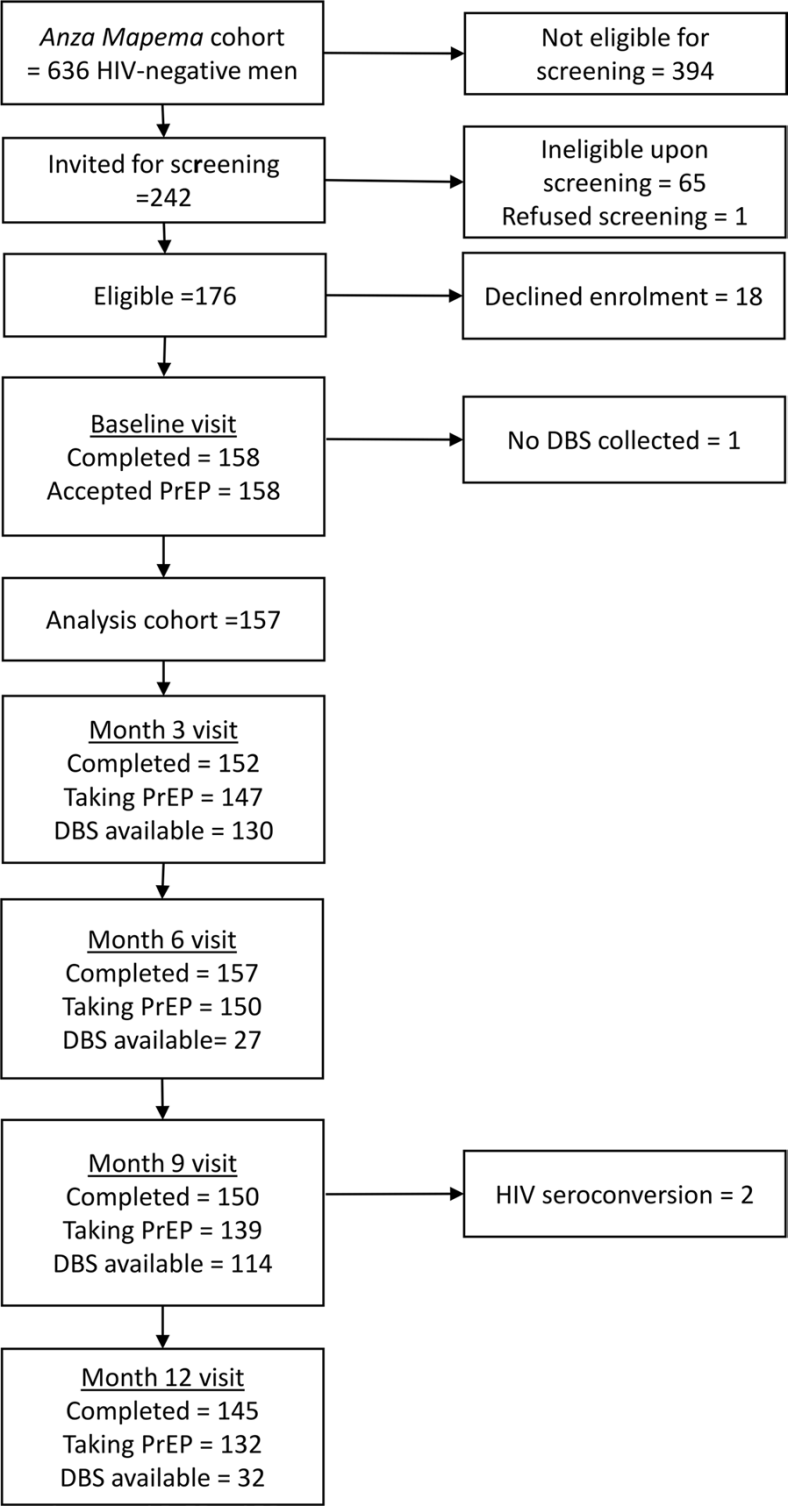
Flow diagram depicting *Anza Mapema Mbili* cohort screening, enrolment, PrEP uptake, visit attendance, PrEP continuation, and DBS collection at each study visit. *Anza Mapema* participants were invited for screening if they reported behavior that met *Anza Mapema Mbili* eligibility criteria at their last cohort visit. If a participant had missed a month 3 or month 9 specimen collection for any reason, DBS were collected at month 6 or month 12 instead

Figure 1 presents details on screening, enrolment, PrEP uptake, visit attendance, PrEP continuation, and DBS collection at each study visit. Of 176 GBMSM who were eligible upon screening, 158 enrolled and completed a baseline visit. All 158 accepted a 30-day PrEP supply at baseline. DBS were collected from 130 participants at month 3, 27 participants at month 6, 114 participants at month 9, and 32 participants at month 12. Overall, 157 (100%) participants provided DBS at 303 visits, with 14 (8.9%) providing 1 sample, 140 (89.2%) providing 2 samples, and 3 (1.9%) providing 3 samples. One participant never had a DBS collected during follow-up and was therefore excluded from further analysis.

Table 1 presents baseline characteristics of the 157 participants with at least one DBS measure. Median age was 27 years (interquartile range, 24–31 years). Overall, 147 (94.2%) reported sex with a male partner, 81 (51.9%) reported sex with a female partner, 108 (69.2%) reported transactional sex, and 98 (62.4%) reported condomless anal sex with a male partner, all in the last 3 months. Nine (5.7%) reported injection drug use in the last 12 months. Participants’ self-assessed chance of acquiring HIV was none for 18.5%, small for 27.4%, moderate for 20.4%, great for 12.1%, and 21.7% did not respond.

**Table 1:** Baseline characteristics of 157 participants and generalized estimating equations analysis of factors associated with protective TFV-DP levels during follow-up

Variable	Baseline characteristics n (Column %)	Unadjusted odds ratio (95% CI)	p value	Adjusted Odds Ratio^ (95% CI)	p value
Age (years)			0.04		0.12
20–24	49 (31.2)	Reference		Reference	
25–29	52 (33.1)	1.28 (0.35–4.74)		1.12 (0.24–5.12)	
≥ 30	56 (35.7)	3.63 (1.17–11.3)		3.27 (0.91–11.7)	
Education (years)			0.08		0.57
Primary (0–8)	36 (22.9)	Reference		Reference	
Secondary (9–12)	79 (50.3)	0.33 (0.12–0.91)		0.65 (0.15–2.71)	
Post-secondary (≥ 13)	42 (26.8)	0.37 (0.11–1.23)		0.39 (0.07–2.21)	
Occupation			0.20		
Unemployed	19 (12.1)	Reference			
Sex worker	17 (10.8)	0.72 (0.21–2.50)			
Casual laborer	29 (18.5)	1.86 (0.73–4.74)			
Employed	92 (58.6)	0.90 (0.32–2.51)			
Marital status			0.53		
Single	106 (67.5)	Reference			
Married/living with female	26 (16.6)	1.43 (0.46–4.39)			
Separated/divorced from female	25 (15.9)	1.86 (0.59–5.84)			
Currently living with a male partner			0.69		
No	108 (68.8)	Reference			
Yes	49 (31.2)	0.83 (0.34–2.05)			
Sex with a male partner (last 3 months)^a^			0.25		
No	9 (5.8)	Reference			
Yes	147 (94.2)	3.09 (0.46–20.8)			
Number of male sex partners (last 3 months)^a^			0.60		
None	9 (5.8)	Reference			
One	41 (26.3)	2.46 (0.35–17.3)			
Two	59 (37.8)	3.54 (0.46–27.3)			
Three or more	47 (30.1)	3.73 (0.47–29.8)			
Sex with a female partner (last 3 months)^a^			0.03		0.16
No	75 (48.1)	Reference		Reference	
Yes	81 (51.9)	2.89 (1.08–7.70)		1.96 (0.77–5.01)	
Number of female sex partners (last 3 months)^a^			0.09		
None	75 (48.1)	Reference			
One	43 (27.6)	3.06 (1.04–8.96)			
Two	17 (10.9)	3.54 (1.10–11.4)			
Three or more	21 (13.5)	1.51 (0.34–6.67)			
Transactional sex (last 3 months)^a^			0.90		
No	48 (30.8)	Reference			
Yes	108 (69.2)	1.06 (0.38–2.94)			
Always used condom for anal intercourse with a man (last 3 months)			0.47		
No	98 (62.4)	Reference			
Yes	59 (37.6)	1.09 (0.44–2.71)			
Not applicable	0	3.35 (0.49–23.1)			
Usual position during sex with a man			0.16		
Insertive	88 (56.0)	Reference			
Receptive	25 (15.9)	1.42 (0.44–4.51)			
Versatile	44 (28.0)	2.34 (0.96–5.71)			
Injection drug use (last 12 months)			0.01		0.02
No	148 (94.3)	Reference		Reference	
Yes	9 (5.7)	5.06 (1.41–18.2)		7.05 (1.31–38.1)	
Sharing needles (last 12 months)^a^			0.93		
No	151 (96.8)	Reference			
Yes	5 (3.2)	1.09 (0.15–7.99)			
Harmful alcohol use (AUDIT > 8)			0.93		
No	84 (53.5)	Reference			
Yes	73 (46.5)	0.96 (0.39–2.37)			
Harmful substance use (DAST > 3)^b^			0.17		
No	116 (74.8)	Reference			
Yes	39 (25.2)	1.95 (0.75–5.04)			
Moderately severe/severe depressive symptoms (PHQ-9 > 15)			0.30		
No	144 (91.7)	Reference			
Yes	13 (8.3)	1.77 (0.60–5.22)			
Social support score greater than median			0.05		0.08
No	84 (53.5)	Reference		Reference	
Yes	73 (46.5)	0.39 (0.15–0.99)		0.41 (0.15–1.13)	
Upsetting sexual experiences during childhood			0.70		
No	106 (67.5)	Reference			
Yes	51 (32.5)	1.20 (0.47–3.08)			
Any verbal or physical abuse, forced sex, or threats (last 3 months)^c^			0.64		
No	84 (57.9)	Reference			
Yes	61 (42.1)	1.25 (0.49–3.20)			
Any disclosure of same-sex behavior to family, friends, at work or school, or in public^d^			0.75		
No	133 (86.4)	Reference			
Yes	21 (13.6)	1.20 (0.38–3.74)			
What do you think are your chances of getting HIV/AIDS?			0.06		0.04
No chance at all	29 (18.5)	Reference		Reference	
Small chance	43 (27.4)	1.10 (0.32–3.78)		2.39 (0.47–12.2)	
Moderate chance	32 (20.4)	3.77 (1.22–11.7)		10.2 (2.01–52.2)	
Great chance	19 (12.1)	1.68 (0.26–10.6)		2.17 (0.28–16.7)	
Don’t know/no response	34 (21.7)	0.51 (0.08–3.17)		0.77 (0.05–12.3)	
Time since enrolment, in months		0.88 (0.80–0.96)	0.006	0.85 (0.74–0.98)	0.02

*AIDS* acquired immunodeficiency syndrome; *AUDIT* Alcohol Use Disorders Identification Test; *CI* confidence interval; *DAST* Drug Abuse Screening Test; *HIV* human immunodeficiency virus; *PHQ-9* = Personal Health Questionnaire 9; TFV-*DP* tenofovir diphosphate

^a^Data were missing for one participant at baseline

^b^Data were missing for two participants at baseline

^c^Data were missing for 12 participants at baseline

^d^Data were missing for three participants at baseline

^N = 154 participants were in the multivariable model

Retention in the cohort was 152 of 157 (96.8%) at month 3, 157 of 157 (100%) at month 6, and 150 of 157 (95.5%) at month 9. Two participants tested positive for HIV at month 9 and were referred for HIV care. At month 12, 145 of the remaining 155 participants (93.5%) were retained (Fig. 1). Overall, risk remained high across visits (Supplemental Table), with no significant decrease in the proportion of participants who reported ongoing HIV risk due to multiple partners, transactional sex, inconsistent condom use or injection drug use. Self-perceived HIV risk increased over time, as fewer participants stating they had “no chance at all” of acquiring HIV across visits (z = 2.82, p = 0.005).

While most participants continued to receive PrEP refills at the clinic after enrollment, self-reported continuation of PrEP decreased over time, with 98.0% continuing at month 3, 95.5% at month 6, 92.7% at month 9, and 91.7% at month 12 (Supplemental Table, z = − 2.61, p = 0.009). Any TFV-DP was detected in 103 (34.0%) samples among 73 (46.5%) individuals; levels were protective in only 29 (9.6%) samples and 23 (14.6%) individuals. In GEE analysis of having protective TFV-DP levels at given visit (Table 1), age, education level, sex with a female partner (yes/no or numbers of female partners), intravenous drug use, social support above the median, HIV risk perception, and time since enrolment were associated with the outcome at p < 0.10. In multivariable analysis, protective TFV-DP levels during follow-up were associated with injection drug use (aOR 7.05, 95% CI 1.31–38.1), and with reporting a self-assessed moderate risk of acquiring HIV (aOR 10.2, 95% CI 2.01–52.2). In addition, the odds of protective TFV-DP levels during follow-up were lower with increasing time since enrolment (aOR 0.85, 95% CI 0.74–0.98 per month).

Self-reported PrEP adherence over study visits was relatively high and stable over follow-up. Only the question “In general, how often do you take your PrEP?” showed a decreasing trend that approached significance, with fewer participants reporting they took their PrEP “all of the time” (z = − 1.74, p = 0.08, details in Supplemental Table). Table 2 presents associations between each self-report adherence measure and both protective TFV-DP level as a binary outcome and TFV-DP levels as a continuous variable. The only self-report adherence measure that was associated with the TFV-DP measures was the question “Rate your ability to take your PrEP as prescribed in the past month,” for which each increment in rating (from “very poor” up through “excellent”) was associated with a 1.79-fold increased odds of protective TFV-DP levels (Χ^2^ = 16.5, p < 0.0001) and rating levels were positively correlated with TFV-DP levels (ρ = 0.172, p = 0.004). Of the two seroconverters, the first reported missing no doses, a “good” ability to take PrEP, which was taken “most of the time,” and a VAS score of 98%; this participant had one DBS at month 6 with undetectable TFV-DP levels. The second seroconverter reported missing PrEP for 3 days in the past month, “poor” ability to take PrEP, which was taken “some of the time,” and a VAS score of 45%; this individual had a DBS at month 3 with protective TFV-DP levels, but their DBS measure at month 9 had undetectable TFV-DP levels.

**Table 2:** GEE analysis of associations between each self-report adherence measure and protective TFV-DP levels and Spearman correlations between each self-report adherence measure and TFV-DP level as a continuous measure at 284 visits on which men reported they were still taking PrEP and had DBS tested

Self-report adherence measure	Odds ratio (95% CI)	Wald Χ^2^-square (p value)	Spearman correlation (p value)
Took PrEP within past day	2.17 (0.79–5.95)	2.28 (0.13)	0.084 (0.16)
Missed taking any PrEP, past month^a^	0.63 (0.30–1.37)	1.35 (0.24)	− 0.055 (0.36)
Proportion of PrEP doses taken, past month (median [IQR])^a^	1.01 (0.99–1.04)	2.11 (0.15)	0.104 (0.08)
Rate your ability to take your PrEP as prescribed, past month^a^*	1.79 (1.35–2.38)	16.5 (< 0.0001)	0.172 (0.004)
In general, how often do you take your PrEP?^a^*	1.47 (0.86–2.49)	2.01 (0.16)	0.058 (0.33)
Visual analog scale (median [IQR])^a^	1.02 (0.97–1.06)	0.62 (0.43)	0.039 (0.51)

*DBS* dried blood spots; *GEE* generalized estimating equations; *PrEP* pre-exposure prophlyaxis; *TFV-DP* tenofovir diphosphate

*These two self-rating scales were analyzed as continuous ordinal predictors, given empty cells for some categories. Details of responses at each study visit are in Supplemental Table 1

^a^Data were missing for 2 participants

Fifty GBMSM community participants and eight researchers (US and Kenyan GBMSM and allies) attended the community charrette. Thematic representations of barriers and facilitators elicited during the charrette at each step of the PrEP cascade (i.e., PrEP uptake, PrEP adherence) are mapped in Table 3. Factors related to lack of information or misinformation; trust in providers or facilities; and stigma and discrimination were barriers to both cascade steps. In addition, barriers included concerns about PrEP safety, efficacy, and side effects; low perceived risk of HIV; poor social support and concerns about disclosure; and lack of autonomy and motivation. Not feeling personal motivation to take PrEP or feeling pressured to enroll in PrEP programming, concerns about partner support or disclosure, and myths and stigma related to PrEP (e.g., that you must take it forever) were among factors that hindered uptake and adherence. In addition, participants mentioned having been motivated to start PrEP for study incentives, and concerns about mixing PrEP with alcohol and other substances. In terms of facilitators influencing participation in the PrEP cascade, tailored information from trusted individuals, information in accessible language, and provider- and clinic-related factors that made access easy and increased trust were identified as important factors. Additional factors that could facilitate PrEP uptake and adherence included GBMSM-friendly services, social support, and feeling personally motivated. In our debrief meeting with the five peer leaders, they emphasized that accurate and non-stigmatizing information about PrEP, PrEP provision in GBMSM-friendly service settings, individualized support for coping with disclosure and stigma, open community discussions around PrEP, and framing PrEP as just one of several options to reduce HIV risk would be critical elements of a successful PrEP support intervention.

**Table 3:** Barriers and facilitators to PrEP uptake, and adherence elicited during the community charrette

	PrEP uptake	PrEP adherence
Barriers	*Information-related factors* • Misinformation or lack of information • Hearing negative things about PrEP • Distortion of information • Lack of information in rural areas • Fear of the unknown *Provider- and clinic-related factors* • Bad provider attitudes and interactions • Perceived lack of confidentiality by staff • Unsafe facilities • Poor provider competence • Distance to facility, cost of transport *Stigma- and discrimination-related factors* • Self-stigma related to HIV • Stigmatization among partners, family, friends • Stigma that PrEP = promiscuity • Stigma and discrimination about HIV/PrEP as ARVs *Pill-related factors* • Concerns about daily dosing • Pill size, taste, odor • Packaging of the drug (like ARV) • Dislike of medications • Concern about taking "forever" • Not wanting to take medicine when you are healthy/not sick *Concerns about safety, efficacy, side effects* • Concerns about safety • Fear of side effects, including liver and kidney toxicity *Low perceived risk* • Feeling that you are not at risk *Social support and disclosure* • Fears about disclosure and storing/keeping meds • Lack of partner support • Fear of disclosure to partners • Fear of IPV *Autonomy and motivation* • Poor "ownership"/feeling of coersion • Poor motivation *Preference for other prevention methods* • Lack of protection from STI • Preference for other prevention measures • Need for condoms to prevent STIs and pregnancy *Don’t want to get tested for HIV* • Fear of HIV testing/screening process	*Information-related factors* • Educational needs/inadequate information • Myths/conceptions: weight gain • Language barriers • Negative influence from other people *Provider- and clinic-related factors* • Availability of PrEP/pill stock-outs • Frequent appointments and long wait times • Breach of confidentiality • Contraindications (provider stops) • Distance to facility, cost of transport • Unfriendly providers or other staff • Distrust from past failed projects/studies • Unable to attend clinic due to work or school schedule *Stigma- and discrimination-related factors* • Focus on risk assessment/sexual orientation • HIV stigma • PrEP stigma *Pill-related factors* • Daily dosing • Pill size • Packaging of the drug (like ARV) • Pill burden *Concerns about safety, efficacy, side effects* • Side effects • Fear of interaction with other medications • Stories of seroconversion • Concerns about use with other substances (alcohol, marijuana, etc.) *Low perceived risk* • Feeling that you are less at risk *Social support and disclosure* • Lack of support system/buddies *Autonomy and motivation* • Funder focus on high enrolment numbers instead of community needs • Incentives as the wrong reason to start *Individual factors* • Failure to be consistent, forgetting • Frequent moves, hard to get refills
Facilitators	*Information-related factors* • Accessible/good information and IEC materials • Awareness creation/community outreach • PrEP champions and peer educators • Testimonials from guys on PrEP, success stories • Theatre, drama, film *Provider- and clinic-related factors* • Existence of GBMSM-friendly facilities • Availability of free PrEP and PrEP services (including HIV testing) • Friendly staff • Trained and qualified providers • Proper guidance and counselling • Support from providers • Less frequent visits, providing extra doses when travel • Financial support for PrEP programming • Reimbursement for transport, food, etc • Location of the facilities *Stigma- and discrimination-related factors* • Destigmatization *Social support and disclosure* • Support groups or PrEP clubs • Support from partners, including HIV+ partners • Drop-in centers for youth or GBMSM • Hotlines, other support services • Other social support (family, friends, etc.) *Autonomy and motivation* • Risk perception • Feeling safe because of PrEP • Testing negative as a motivation	*Information-related factors* • Accessible/good information and IEC materials • Advocacy around combination HIV prevention and sexual/reproductive health • Technical working groups • Outreaches, sensitization • Testimonials from guys on PrEP, success stories • PrEP champions • Social media, ads • Theatre, drama, film *Provider- and clinic-related factors* • Availability of free PrEP and PrEP services (including HIV testing) • Integration of PrEP services at public facilities • Comprehensive services at one point near community • Trained and qualified providers • Support from providers • Perceived confidentiality in the facility • Continuous follow-up and reminders • Community PrEP dispensing • Transportation allowance/"motivation" • New interventions (injectable, long-acting tablets, better tasting pills) *Pill-related factors* • Only one pill per day *Concerns about safety, efficacy, side effects* • Fewer side effects *Social support and disclosure* • Support groups or PrEP clubs • Support from partners, including HIV+ partners • Other social support (family, friends, etc.) *Autonomy and motivation* • Risk perception • Sex work as a motivator • Feeling safe because of PrEP • Testing negative as a motivation • Sexual pleasure and value in sex life • Having an HIV-positive partner • Stories of seroconversion *Individual factors* • Use of reminders (alarms, SMS, etc.)

*ARV* antiretroviral medications; *GBMSM* gay, bisexual, or other men who have sex with men; *HIV* human immunodeficiency virus; *IEC* information, education, and communication; *IPV* intimate partner violence; *PrEP* pre-exposure prophylaxis; *SMS* short message service; *STI* sexually transmitted infection

## Discussion

We report the results of a mixed-methods sequential explanatory design study that involved an initial observational cohort designed to evaluate PrEP uptake and adherence in a real-world, practice-based setting in the initial period after PrEP became available to key populations in Kenya. PrEP uptake among the 176 eligible men offered participation was relatively high, as 158 men (90%) enrolled and accepted an initial 30-day supply of tenofovir-emtricitabine. In addition, retention in the cohort was high, with > 93% of participants attending each visit. Although HIV risk in the cohort remained high and perceived risk increased somewhat, several participants discontinued PrEP during follow-up. Despite high self-reported adherence that did not change significantly over time, protective TFV-DP levels were present in one or more DBS samples from only 14.6% of participants and were less frequent as time from PrEP uptake increased. A community charrette conducted in the second phase of the study with members and service providers from the GBMSM community revealed many important barriers to PrEP uptake and adherence that seem to have outweighed the facilitators at this early point in the PrEP roll-out.

Our results suggest that an affirming clinic environment and basic person-facing adherence support were not sufficient to promote consistent and sustained use of PrEP for much of the cohort population, despite ongoing HIV risk. Of note, a prior analysis of data from this cohort also found no change in sexual practices or incidence of chlamydia and gonorrhea across visits, and no association between either self-reported PrEP adherence or TFV-DP detection and sexual behavior over time [37]. Our finding in the current analysis that reporting injection drug use, a very high-risk behavior, and HIV risk perception (specifically, a self-perceived moderate risk of HIV acquisition) were associated with protective TFV-DP levels is encouraging, and suggests that some participants valued PrEP as an effective prevention tool. Of note, self-assessed risk of HIV acquisition was not associated with any of the sexual behaviors assessed for this study at baseline or over follow-up (details not shown). Interventions that assist participants in accurately assessing their risk for HIV infection may be helpful as a way to encourage GBMSM to initiate and adhere to PrEP for protection against HIV acquisition during periods of risk. It remains to be seen whether interventions can build on these insights to improve PrEP uptake and adherence for Kenyan GBMSM at high risk for HIV acquisition.

Results from other early studies of PrEP among Kenyan GBMSM, some of which also included transgender women, have similarly identified challenges with PrEP adherence. Among GBMSM participants in a Mombasa area cohort, TFV-DP levels in DBS were low, with only 14.5% having protective TFV-DP levels compatible with ≥ 4 doses a week [16]. In a smaller study in the coastal town of Malindi, Kenya, none of the 34 participating GBMSM for whom DBS were collected had protective TFV-DP levels [38]. When combined with evidence that opportunities to screen GBMSM for PrEP eligibility and to help them initiate PrEP are frequently missed in Kenya [39], these data suggest that GBMSM have derived little benefit from PrEP to date. Data are lacking from other African countries, as we were unable to identify any additional published studies of biomarker-based PrEP adherence that included African GBMSM other than the iPrEx trial [3].

In our study, we used several self-report measures of adherence that had been validated in studies of persons living with HIV infection [30–32]. Relatively high self-reported adherence, especially with the visual analog scale [32], and a hesitancy of our study participants to discuss their doubts about PrEP led us to believe that adherence was higher than it actually was. In our analysis of associations between these self-reported measures and objective biomarker measures of PrEP adherence, two of the measures stood out: self-reported frequency of taking PrEP as prescribed trended lower over follow-up, and self-reported ability to take PrEP correlated significantly with protective TFV-DP levels and with TFV-DP levels as a continuous measure. While some men felt comfortable reporting that they had stopped using PrEP or were struggling with adherence, others likely reported good adherence due to social desirability bias. Stigma, lack of trust in providers, and problems with comprehension of the measures used may have also played a role in over-reporting PrEP adherence. Research to improve the performance of self-reported adherence measures among vulnerable populations such as GBMSM in rights-constrained settings may be needed. Regardless, discrepancies between self-report and biomarker-based adherence have been reported even in well-resourced settings [40], and therefore biomarker-based measures are an important standard for evaluation of interventions, such as PrEP, for which adherence is critical to efficacy. Simple-to-use point-of-care urine tests to assess PrEP adherence are emerging and may prove useful in clinical practice [41, 42], especially since testing of DBS samples is expensive and not widely available. Unfortunately, because we shipped and batch-tested our DBS samples at the end of the study for practical reasons, we were unable to adapt study procedures in real time in response to the very low TFV-DP levels detected.

Because we had limited qualitative data from our cohort to explain our DBS results, we implemented a qualitative explanatory phase to explore barriers and facilitators in the PrEP cascade that could help explain our quantitative findings, and would also inform a subsequent funded project to develop a PrEP support intervention for this population. Our findings from the community charrette with members of local grassroots GBMSM organizations and our debrief meeting with GBMSM peer leaders identified key areas of focus for future programs focused on PrEP uptake and adherence for Kenyan GBMSM:

1. Accurate, non-stigmatizing information about PrEP from multiple stakeholders (providers, other GBMSM, partners, media) that dispels myths and misconceptions about PrEP.
2. PrEP provision at GBMSM-friendly clinics with non-stigmatizing, trustworthy providers and staff, ideally including clinicians and counselors who identify as members of the GBMSM community.
3. Individualized peer support from other GBMSM, including assistance with disclosure of PrEP use to partners and other important people, and strategies for PrEP adherence and for coping with GBMSM- and PrEP-related stigma.
4. Opportunities to connect with other GBMSM in group settings, such as through PrEP clubs or drop-in centers, to facilitate community discussions on sexual health.
5. Provision of supportive HIV prevention services to GBMSM whether or not they decide to take PrEP, to reduce any feelings of coercion and ensure that PrEP use is matched to risk.

While some of the barriers and facilitators we identified in the charrette have been noted by others working with GBMSM in Kenya [16, 38], our community charrette and peer debrief meeting demonstrated more clearly than prior research how community consultation and partnership can provide important insights to help address the health concerns of GBMSM in rights-constrained settings.

This study had several limitations. First, the cohort sample was small and not representative of all GBMSM in Kenya, reducing generalizability. Second, most of the measures were based on self-report, which is prone to recall limitation and social desirability biases. Third, we were only able to measure biomarkers of PrEP adherence at two time points due to limited funding, and had missing DBS at one or more time points for several participants despite high overall retention. Fourth, while counseling messages were standardized and a basic grounding in motivational interviewing approaches was provided to all research counselors at baseline, counseling quality was not monitored. Finally, we did not conduct in-depth interviews with cohort participants at the time of study withdrawal, PrEP discontinuation, or study completion, and so are inferring indirectly from our community charrette that the barriers identified were present for cohort participants. Despite these limitations, the inclusion of PrEP drug levels and of insights from the GBMSM community are important strengths. This study adds to growing evidence that there are multiple challenges to PrEP adherence among GBMSM, and that additional work is needed in Kenya and other rights-constrained settings to ensure that GBMSM can benefit from this proven HIV prevention tool.

In summary, despite relatively high self-reported adherence to PrEP in this cohort of Kenyan GBMSM, actual TFV-DP levels were protective in only 14.6% of participants at any visit and decreased use over follow-up despite ongoing risk. Although participants reporting injection drug use and moderate perceived risk of HIV acquisition had better adherence than other participants, adherence in these individuals was still very low and was not sustained over time. A community charrette with 50 GBMSM participants revealed numerous barriers to PrEP uptake and adherence, as well as facilitators that could be leveraged to improve outcomes. GBMSM community participation in promotion and delivery of PrEP and in the development of interventions to address the barriers to and facilitators identified in this research will be critical to improving HIV prevention services, including PrEP delivery, for this vulnerable population.

## Supplementary Information

Below is the link to the electronic supplementary material.

Supplementary file1 (DOCX 33 KB)

## Data Availability

De-identified cohort data will be deposited in the Harvard Dataverse (https://dataverse.harvard.edu/) upon publication.

## References

[1] Beyrer C, Baral SD, Collins C (2016). The global response to HIV in men who have sex with men. Lancet.

[2] Beyrer C, Baral SD, van Griensven F (2012). Global epidemiology of HIV infection in men who have sex with men. Lancet.

[3] Grant RM, Lama JR, Anderson PL (2010). Preexposure chemoprophylaxis for HIV prevention in men who have sex with men. N Engl J Med.

[4] Anderson PL, Glidden DV, Liu A (2012). Emtricitabine-tenofovir concentrations and pre-exposure prophylaxis efficacy in men who have sex with men. Sci Transl Med..

[5] World Health Organization (2015). Guideline on when to start antiretroviral therapy and on pre-exposure prophylaxis for HIV.

[6] Donnell D, Baeten JM, Bumpus NN (2014). HIV protective efficacy and correlates of tenofovir blood concentrations in a clinical trial of PrEP for HIV prevention. J Acquir Immune Defic Syndr.

[7] World Health Organization (2014). Consolidated guidelines on HIV prevention, diagnosis, treatment and care for key populations.

[8] Ministry of Health (2014). Kenya HIV prevention revolution road map: Countdown to 2013.

[9] Masyuko S, Mukui I, Njathi O (2018). Pre-exposure prophylaxis rollout in a national public sector program: the Kenyan case study. Sex Health.

[10] Ministry of Health (2017). Framework for the implementation of pre-exposure prophylaxis of HIV in Kenya.

[11] Mutua G, Sanders E, Mugo P (2012). Safety and adherence to intermittent pre-exposure prophylaxis (PrEP) for HIV-1 in African men who have sex with men and female sex workers. PLoS ONE.

[12] Van der Elst EM, Mbogua J, Operario D (2013). High acceptability of HIV pre-exposure prophylaxis but challenges in adherence and use: qualitative insights from a phase I trial of intermittent and daily PrEP in at-risk populations in Kenya. AIDS Behav.

[13] Mugo PM, Sanders EJ, Mutua G (2015). Understanding adherence to daily and intermittent regimens of oral HIV pre-exposure prophylaxis among men who have sex with men in Kenya. AIDS Behav.

[14] Karuga RN, Njenga SN, Mulwa R, Kilonzo N, Bahati P, O’Reilley K, Gelmon L, Mbaabu S, Wachichi C, Githuka G, Kiragu M (2016). “How I wish this thing was initiated 100 years ago!” Willingness to take daily oral pre-exposure prophylaxis among men who have sex with men in Kenya. PLoS ONE.

[15] Wahome E, Graham S, Thiong’o A, Chirro O, Mohamed K, Gichuru E, Mwambi, Prince M, Sanders EJ (2020). Assessment of PrEP eligibility and uptake among at-risk MSM participating in a HIV-1 vaccine feasiility cohort in coastal Kenya. Welcome Open Res..

[16] Wahome EW, Graham SM, Thiong'o AN (2020). PrEP uptake and adherence in relation to HIV-1 incidence among Kenyan men who have sex with men. EClinicalMedicine.

[17] Kimani M, van der Elst EM, Chiro O (2019). PrEP interest and HIV-1 incidence among MSM and transgender women in coastal Kenya. J Int AIDS Soc.

[18] Wahome EW, Graham SM, Thiong’o AN, Mohamed K, Oduor T, Gichuru E, Mwambi J, van der Elst EM, Sanders EJ (2020). Risk factors for loss to follow-up among at-risk HIV negative men who have sex with men participating in a research cohort with access to pre-exposure prophylaxis in coastal Kenya. J Int AIDS Soc.

[19] Kunzweiler CP, Bailey RC, Okall DO (2017). Factors associated with prevalent HIV infection among Kenyan MSM: the Anza Mapema study. J Acquir Immune Defic Syndr.

[20] Ministry of Health (2015). Guidelines for HIV testing services in Kenya.

[21] Graham SM, Micheni M, Kombo B (2015). Development and pilot testing of an intervention to promote care engagement and adherence among HIV-positive Kenyan MSM. AIDS.

[22] Graham SM, Micheni M, Chirro O (2020). A randomized controlled trial of the Shikamana intervention to promote antiretroviral therapy adherence among gay, bisexual, and other men who have sex with men in Kenya: feasibility, acceptability, safety and initial effect size. AIDS Behav.

[23] Kizub D, Quilter L, Atieno L (2020). Challenges faced by peer outreach workers in an HIV prevention and care program for gay, bisexual, and other men who have sex with men: the Anza Mapema Study. Glob J Commun Psychol Practice..

[24] Saunders JB, Aasland OG, Babor TF (1993). Development of the alcohol use disorders identification test (AUDIT): WHO collaborative project on early detection of persons with harmful alcohol consumption—II. Addiction.

[25] Skinner HA (1982). The drug abuse screening test. Addict Behav.

[26] Kroenke K, Spitzer RL, Williams JB (2001). The PHQ-9: validity of a brief depression severity measure. J Gen Intern Med.

[27] Sherbourne CD, Stewart AL (1991). The MOS social support survey. Soc Sci Med.

[28] Smith N, Lam D, Bifulco A, Checkley S (2002). Childhood Experience of Care and Abuse Questionnaire (CECA.Q). Validation of a screening instrument for childhood adversity in clinical populations. Soc Psychiatry Psychiatr Epidemiol.

[29] Betron M, Gonzalez-Figueroa E (2009). Gender identity and violence in MSM and transgenders: policy implications for HIV services.

[30] Wilson IB, Lee Y, Michaud J (2016). Validation of a new three-item self-report measure for medication adherence. AIDS Behav.

[31] Wilson IB, Fowler FJ, Cosenza CA (2014). Cognitive and field testing of a new set of medication adherence self-report items for HIV care. AIDS Behav.

[32] Oyugi JH, Byakika-Tusiime J, Charlebois ED (2004). Multiple validated measures of adherence indicate high levels of adherence to generic HIV antiretroviral therapy in a resource-limited setting. J Acquir Immune Defic Syndr.

[33] National AIDS Control Council (2018). Kenya HIV estimates report 2018.

[34] Klamerus ML, Damschroder LJ, Sparks JB (2019). Developing strategies to reduce unnecessary services in primary care: protocol for user-centered design charrettes. JMIR Res Protoc.

[35] Braun V, Clarke V (2006). Using thematic analysis in psychology. Qual Res Psychol.

[36] Nowell LS, Norris JM, White DE, Moules NJ (2017). Thematic analysis: striving to meet the trustworthiness criteria. Int J Qual Methods.

[37] Mehta SD, Okall D, Graham SM, Ngety G, Bailey RC, Otieno F (2021). Behavior change and sexually transmitted incidence in relationship to PrEP use among men who have sex with men in Kenya. AIDS Behav.

[38] Kimani M, van der Elst EM, Chirro O, Wahome E, Ibrahim F, Mukuria N, de Wit TFR, Graham SM, Operario D, Sanders EJ (2021). “I wish to remain HIV negative”: pre-exposure prophylaxis adherence and persistence in transgender women and men who have sex with men in coastal Kenya. PLoS ONE.

[39] Were D, Musau A, Mutegi J (2020). Using a HIV prevention cascade for identifying missed opportunities in PrEP delivery in Kenya: results from a programmatic surveillance study. J Int AIDS Soc.

[40] Hebel S, Kahn-Woods E, Malone-Thomas S (2020). Discrepancies between self-reported adherence and a biomarker of adherence in real-world settings. J Acquir Immune Defic Syndr.

[41] Gandhi M, Bacchetti P, Spinelli MA (2019). Brief report: Validation of a urine tenofovir immunoassay for adherence monitoring to PrEP and ART and establishing the cutoff for a point-of-care test. J Acquir Immune Defic Syndr.

[42] Drain P, Ngure K, Mugo N (2020). Testing a real-time tenofovir urine adherence assay for monitoring and providing feedback to preexposure prophylaxis in Kenya (PUMA): Protocol for a pilot randomized controlled trial. JMIR Res Protoc.

