# Supplementary material for: Challenges with PrEP Uptake and Adherence Among Gay, Bisexual, and Other Men Who Have Sex with Men in Kisumu, Kenya
Source: AIDS Behav. 2022 Oct 11;27(4):1234–47. doi: 10.1007/s10461-022-03860-w (PMC10036412; doi:10.1007/s10461-022-03860-w)
Supplement: Supplementary file 1 — Supplementary file1 (DOCX 33 KB) [file 10461_2022_3860_MOESM1_ESM.docx]

**Supplemental Table 1. Risk behaviors and risk perception over follow-up visits**

| *Variable* | *Month 3*  *N = 152* | *Month 6*  *N = 157* | *Month 9*  *N = 150* | *Month 12*  *N = 145* | *Z score (P value)*  *for trend** |
| --- | --- | --- | --- | --- | --- |
| Sex with a male partner (last 3 months) ^a^ | 130 (89.7) | 129 (86.0) | 127 (87.0) | 119 (87.5) | -0.45 (0.65) |
| Number of male sex partners (last 3 months) ^a^  None  One  Two  Three or more | 15 (10.3)  49 (33.8)  54 (37.2)  27 (18.6) | 21 (14.0)  61 (40.7)  37 (24.7)  31 (20.7) | 19 (13.0)  56 (38.4)  48 (32.9)  23 (15.8) | 17 (12.5)  58 (42.6)  38 (27.9)  23 (16.9) | -1.38 (0.17) |
| Sex with a female partner (last 3 months) ^b^ | 74 (51.0) | 79 (51.0) | 70 (47.3) | 65 (46.4) | -0.94 (0.35) |
| Number of female sex partners (last 3 months) ^b^  None  One  Two  Three or more | 71 (49.0)  31 (21.4)  28 (19.3)  15 (10.3) | 76 (49.0)  35 (22.6)  28 (18.1)  16 (10.3) | 78 (52.7)  31 (21.0)  23 (15.5)  16 (10.8) | 75 (53.6)  28 (20.0)  19 (13.6)  18 (12.9) | -0.75 (0.45) |
| Transactional sex (last 3 months) ^c^ | 104 (68.9) | 108 (69.2) | 105 (70.5) | 101 (70.1) | 0.30 (0.76) |
| Inconsistent condom use for anal intercourse with a man (last 3 months) ^d^ | 69 (46.0) | 83 (52.9) | 81 (54.0) | 68 (47.2) | 0.28 (0.78) |
| Usual position during sex with a man ^e^  Insertive  Receptive  Versatile | 92 (61.7)  26 (17.4)  31 (20.8) | 93 (60.0)  23 (14.8)  39 (25.2) | 92 (62.6)  22 (15.0)  33 (22.4) | 80 (56.7)  28 (19.9)  33 (23.4) | 0.61 (0.54) |
| Injection drug use (last 12 months) | 8 (5.3) | 9 (5.7) | 9 (6.0) | 9 (6.2) | 0.36 (0.72) |
| Sharing needles (last 12 months) ^c^ | 4 (2.6) | 5 (3.2) | 5 (3.4) | 5 (3.5) | 0.41 (0.68) |
| Composite HIV risk † | 133 (94.3) | 138 (92.6) | 133 (93.0) | 121 (90.3) | -1.16 (0.25) |
| What do you think are your chances of getting HIV/AIDS?  No chance at all  Small chance  Moderate chance  Great chance  Don’t know/no response | 49 (32.2)  51 (33.6)  24 (15.8)  8 (5.3)  20 (13.2) | 34 (21.7)  52 (33.1)  25 (15.9)  12 (7.6)  34 (21.7) | 38 (25.3)  54 (36.0)  27 (18.0)  9 (6.0)  22 (14.7) | 27 (18.6)  47 (32.4)  24 (16.6)  5 (3.4)  42 (29.0) | 2.82 (0.005) |
| Still taking PrEP at study visit ^d^ | 147 (98.0) | 150 (95.5) | 139 (92.7) | 132 (91.7) | -2.61 (0.009) |
| Took PrEP within past day ^f^ | 122 (83.0) | 105 (72.9) | 104 (75.9) | 97 (79.5) | -0.57 (0.57) |
| Missed taking any PrEP, past month ^g^ | 47 (32.0) | 71 (49.3) | 57 (42.9) | 46 (39.0) | 0.91 (0.36) |
| Days of PrEP missed, past month ^g^  None  1-7 days  >7 days | 100 (68.0)  36 (24.5)  11 (7.5) | 73 (50.7)  46 (31.9)  25 (17.4) | 76 (57.1)  47 (35.3)  10 (7.5) | 72 (61.0)  37 (31.4)  9 (7.6) | 0.62 (0.53) |
| Proportion of PrEP doses taken, past month (median [IQR]) ^g^ | 100 (96,7–100) | 100 (86.7–100) | 100 (93.3–100) | 100 (93.3–100) | -0.62 (0.53) |
| Rate your ability to take your PrEP as prescribed, past month ^g^  Very poor  Poor  Fair  Good  Very good  Excellent | 1 (0.7)  0  10 (6.8)  44 (29.9)  71 (48.3)  21 (14.3) | 2 (1.4)  3 (2.1)  7 (4.9)  51 (35.4)  66 (45.8)  15 (10.4) | 1 (0.8)  2 (1.5)  7 (5.3)  53 (39.8)  52 (39.1)  18 (13.5) | 4 (3.4)  0  4 (3.4)  35 (29.7)  56 (47.5)  19 (16.1) | 0.11 (0.91) |
| In general, how often do you take your PrEP? ^g^  None of the time  A little of the time  Some of the time  A good bit of the time  Most of the time  All of the time | 0  0  1 (0.7)  12 (8.2)  44 (29.9)  90 (61.2) | 1 (0.7)  0  5 (3.5)  9 (6.2)  43 (29.9)  86 (59.7) | 0  5 (3.8)  2 (1.5)  3 (2.3)  44 (33.1)  79 (59.4) | 0  1 (0.8)  3 (2.5)  7 (5.9)  50 (42.4)  57 (48.3) | -1.74 (0.08) |
| Visual analog scale (median [IQR]) ^g^ | 98 [90–100] | 98 [88.5–100] | 98 [90-100] | 97.5 [90–99] | -0.52 (0.60) |

Abbreviations: AIDS = acquired immunodeficiency syndrome; HIV = human immunodeficiency virus; IQR = interquartile range

* Cuzick’s nonparametric test for trend.

† A composite measure of HIV risk was created to indicate when any of the following was present: two or more partners (male or female), transactional sex, inconsistent condom use with male partners, injection drug use, or needle sharing.

^a^ Data were missing for 27 participants over 606 visits.

^b^ Data were missing for 16 participants over 606 visits.

^c^ Data were missing for four participants over 606 visits.

^d^ Data were missing for three participants over 606 visits.

^e^ Data were missing for 12 participants over 606 visits.

^f^ Data were missing for 18 participants over 568 visits at which men reported they were still taking PrEP.

^g^ Data were missing for 26 participants over 568 visits at which men reported they were still taking PrEP.
